# Response of Moose Hunters to Predation following Wolf Return in Sweden

**DOI:** 10.1371/journal.pone.0119957

**Published:** 2015-04-08

**Authors:** Camilla Wikenros, Håkan Sand, Roger Bergström, Olof Liberg, Guillaume Chapron

**Affiliations:** 1 Grimsö Wildlife Research Station, Department of Ecology, Swedish University of Agricultural Sciences, Riddarhyttan, Sweden; 2 Forestry Research Institute of Sweden, Uppsala Science Park, Uppsala, Sweden; Queen Mary, University of London, UNITED KINGDOM

## Abstract

**Background:**

Predation and hunter harvest constitute the main mortality factors affecting the size and dynamics of many exploited populations. The re-colonization by wolves (*Canis lupus*) of the Scandinavian Peninsula may therefore substantially reduce hunter harvest of moose (*Alces alces*), the main prey of wolves.

**Methodology/Principal findings:**

We examined possible effects of wolf presence on hunter harvest in areas where we had data before and after wolf establishment (n = 25), and in additional areas that had been continuously exposed to wolf predation during at least ten years (n = 43). There was a general reduction in the total number of moose harvested (n = 31,827) during the ten year study period in all areas irrespective of presence of wolves or not. However, the reduction in hunter harvest was stronger within wolf territories compared to control areas without wolves. The reduction in harvest was larger in small (500-800 km^2^) compared to large (1,200-1,800 km^2^) wolf territories. In areas with newly established wolf territories moose management appeared to be adaptive with regard to both managers (hunting quotas) and to hunters (actual harvest). In these areas an instant reduction in moose harvest over-compensated the estimated number of moose killed annually by wolves and the composition of the hunted animals changed towards a lower proportion of adult females.

**Conclusions/Significance:**

We show that the re-colonization of wolves may result in an almost instant functional response by another large predator—humans—that reduced the potential for a direct numerical effect on the density of wolves’ main prey, the moose. Because most of the worlds’ habitat that will be available for future colonization by large predators are likely to be strongly influenced by humans, human behavioural responses may constitute a key trait that govern the impact of large predators on their prey.

## Introduction

The main mortality factors affecting the size and dynamics of many ungulate populations are predation [1, 2, 3] and harvest by hunters [4, 5]. Declining populations of large predators during the 20th century generally resulted in hunter harvest becoming the prime limiting factor. However, in some places, large predators are now re-colonizing areas where hunter harvest for a long time had been the main factor limiting ungulate populations [6, 7]. As large predators often have a major impact on their prey populations [1, 8], predation might have direct implications for the size of sustainable hunter harvest of ungulate populations. Therefore, reductions in hunter harvests may be necessary in the presence of increasing predator populations to avoid declines in exploited ungulate populations [9, 10, 11]. However, the intensity of competition between hunters and large predators will depend on the degree of compensatory mortality of both mortality factors, i.e. if predation to a large extent is compensatory to other causes of mortality (than harvest), hunter harvest will be less influenced by predators than if predation includes a large additive component [12].

The contribution to ungulate population growth generally differs between age and sex classes [13]. Therefore, in extensively harvested ungulate populations management in terms of decided sex ratios and age structures is important [14]. In general, the pattern of harvest mortality differs from mortality by predators regarding the age and sex classes removed. For polygynous species, hunters commonly bias their harvest toward males to maintain a female-biased population with a high net production [15, 16]. Trophy hunting of prime males causes a lower average age of males compared to populations with no hunter harvest [17, 18]. In contrast, predators like African wild dog (*Lycaon pictus*), wolves (*Canis lupus*) and brown bears (*Ursus arctos*) focus on the more vulnerable segments of the prey population, such as young animals or individuals in poor condition [19, 20, 21, 22], whereas lynx (*Lynx lynx*) for example kill prey according to their relative frequency of occurrence [23].

The Scandinavian moose population has been one of the most extensively harvested moose populations in the world since the 1960s [24]. Moose hunting has both great economic and recreational value [25, 26], and provides a considerable amount of meat and income to landowners. There are also considerable costs associated with high moose densities mainly through damage to agricultural crops and forest trees [27] and moose-vehicle collisions [28].

Hunters in Scandinavia are organized into geographically distinct management units that in turn consist of a large number of hunting teams [27]. This means that each unit/team is confined to hunt moose in a specific area on a long term basis. Harvest quotas are normally decided as a result of negotiations between hunters, stakeholders and the authorities who ultimately decide and finalize the quotas but hunting teams may decide themselves to what extent they should fill their given quota for any year. As a consequence, this management system includes an incentive for the hunters to strive for a sustainable harvest in a multi-year perspective, because a harvest larger that the annual sustainable yield inevitably will lead to a reduction in the moose population size and thereby the potential for future harvest.

The re-colonization by wolves on the Scandinavian Peninsula has largely been opposed by many hunters. One of several arguments against wolves has been that this would lead to a significant decline in the moose population and severely reduce the potential for hunter harvest. This view is supported by the fact that moose are the main prey of wolves during both summer and winter [20, 29, 30, 31] and wolf predation is mainly additive to natural mortality [32]. In order to avoid a decline in moose density after wolf establishment hunters must reduce their harvest numerically or change the composition of the hunted animals by reducing the harvest of females [11, 18].

We examined the effect of wolf predation on hunter harvest of moose within wolf territories, and compared this to hunter harvest in adjacent control areas without wolves. The effect of wolf predation on hunter harvest was quantified both during the phase of wolf establishment as well as in areas where wolves had been present continuously during at least ten consecutive years. We predicted that the establishment of a wolf territory would result in a reduction in the number of moose harvested and a change in composition of hunted animals. Specifically, we investigated: 1. if the reduction in hunter harvest compensated for wolf predation; 2. how the composition of the hunted animals changed with the presence of wolves; 3. if the reduction in hunter harvest was larger in small compared to large wolf territories as a result of wolf kills being concentrated in a smaller area; and, 4. if the reduction in harvest could be predicted from a reduction in hunting quotas set prior to harvest.

## Methods

### Study Site and Species

The study was performed in 13 wolf territories with adjacent control areas within six counties (Dalarna, Gävleborg, Värmland, Västmanland, Västra Götaland, and Örebro) in the boreal vegetation zone [33] in south-central Sweden. In the winter of 1994/1995 the wolf population consisted of three packs and one pair (16–20 wolves) [7] and in the winter of 2007/2008 of 17–25 packs and 20–21 pairs (166–210 wolves) [34].

Winter densities of moose within wolf territories ranged between 0.6 and 3.4 moose km^-2^, according to estimates from pellet group counts and aerial censuses [31]. Approximately 100,000 individuals or 25–30% of the pre-harvest moose population were harvested annually in the beginning of the 21st century in Sweden [24]. The harvest season lasts from the second Monday in October (except in some areas in three counties where hunting also was allowed during three weeks in September) until the last day of January or February (in one county).

### Ethics Statement

All procedures including capture, handling and collaring of wolves [35] fulfilled ethical requirements and have been approved by the Swedish Animal Welfare Agency (Permit Number: C 281/6).

### Hunter Harvest of Moose

We used data on the annual number of moose harvested and quotas issued (number of adults (yearlings included) and calves) in management units (average size 60 ± 20 (95% CI) km^2^, range 10–470) obtained from the County Administrative Boards. Each management unit had a quota for a number of hunting teams and all management units are obliged to report the number and category (male, female and calf) of harvested moose each year to the County Administrative Board. For one wolf territory and adjacent control area, the data were received from the major land owner (the forest company Stora Enso).

### Study Design

We used management units where data on hunter harvest were available for each year during a ten-year period between 1995 and 2008. Related to time of wolf establishment (on a yearly basis), harvest rates were examined using two kinds of study areas. We compared harvest data for five years prior to the year of establishment of a wolf pair, and for the first five years after the establishment (hereafter referred to as 5+5 year areas). We only used wolf territories in which a minimum of two wolves (a scent marking pair) were present each year. We used data from all management units (average size 50 ± 10 (95% CI) km^2^, range 10–330) where more than 90% of the area was located within the wolf territory. Data from control areas with no established wolf territories were based on the same number of management units as in areas with wolf occupancy and for the same time period. The same design was used to compare harvest data in areas that have had a continuous presence of wolves for at least ten consecutive years (hereafter referred to as 10-year areas) with control areas. Control areas (average size 80 ± 30 (95% CI) km^2^, range 10–470) were located on average 23 km (range 1–63, n = 68) away from their respective closest wolf territory. This distance was calculated as the distance between the nearest outline of the control area and the nearest border of the wolf territory. To control for differences in size of management units between years and units we used annual quota and number of harvested moose 10 km^-2^ (± 95% CI).

The year of wolf establishment, duration of wolf territories and the geographic distribution of territories for packs and pairs were known due to extensive snow tracking (mainly conducted by the County Administrative Boards), and DNA analyses (conducted by the Wildlife Damage Centre), in combination with VHF/GPS locations from collared wolves, every winter from 1978–1979 to 2007–2008. All location data from snow-tracking and collared wolves during the five- and ten-year periods were pooled and territory size and borders were determined using the 100% minimum convex polygon method (MCP). Reproducing bears occurred in two wolf territories with adjacent control areas in 5+5 year areas (within the counties of Dalarna and Gävleborg) [36]. The establishment of bears occurred before the wolves established in the area.

Harvest data were available for 25 management units (within seven wolf territories) in 5+5 year areas, and for additional 43 management units (within six wolf territories) in 10-year areas. For 10-year areas, the first year used in the analysis was on average three years after establishment of wolves in the area (range 0–15 years). Seven control areas were used in both analyses of 5+5 year areas and 10-year areas.

Data of harvest quotas and number of harvested adult moose were available for 22 management units (within seven wolf territories) in 5+5 year areas, and for 38 management units (within five wolf territories) in 10-year areas with their respective control area. Data on harvest quotas were not available for the total number of moose and calves separately, because calves were exposed to unlimited hunting quotas in some management units.

### Hunting Effort

To test for temporal variation in the effort among hunters we analyzed data on the total number of hours spent hunting for all persons in hunting teams during the first week of the moose hunt in which the major part (up to 75%) of the harvest is conducted [37]. Data were available from the Swedish Association for Hunting and Wildlife Management for 1997–2008. We only used hunting teams that had reported hunting effort during a minimum of six years during the ten year period (this reporting is voluntary). This resulted in available data from 24 hunting teams distributed in five different wolf territories in 5+5 year areas and 23 hunting teams in control areas. In 10-year areas we used data from 45 hunting teams within six different wolf territories and 48 in control areas. On average, data were available during 8 of the 10 study years both in 5+5 year areas and in 10-year areas. In total, for all areas and years, hunters spent 1021,703 hours hunting.

### Hunter Observations

Hunter observations of moose reflect moose density and reproduction reasonably well [38] and may be used to investigate trends over time [39] even in relatively small areas (130 km^2^) [40]. The methodology includes number of observed males, females with zero, one, or two accompanying calves, solitary calves, and unclassified moose by each hunting team during the first week of the moose hunt [38]. As with data on hunting effort we received hunter moose observation data (reported voluntarily by hunting teams) for 1997–2008 from the Swedish Association for Hunting and Wildlife Management, and compiled the number of hours of observation for each hunter team. We pooled all data from hunting teams inside management units used in the analysis of number of harvested moose, and calculated the total number of moose as well as number of males, females, calves, and calves per female observed per hour spent on hunting for each year. We only used years with a minimum of 5,000 hours week^-1^ of observation in both wolf territories and adjacent control areas. This resulted in a total of 34 years with data distributed in four different wolf territories in 10-year areas and a similar number of years in control areas. In total, 707,494 hours of observation by hunters were made and yielded 42,742 observations of moose including 20% males, 43% females, 29% calves and 8% moose of unknown category.

### Analyses

All analyses were conducted using SPSS Statistics 19.0 for Windows (IBM SPSS Inc., Chicago, Illinois, USA). We analysed the annual number of harvested moose 10 km^-2^ (total number of moose as well as number of males, females and calves) to test for differences between wolf territories and control areas (two types; 5+5 year areas and 10-year areas) using a general linear model with repeated measures option (GLM repeated measures). The repeated contrast was used to analyse the effect of time (ten consecutive years) and the interaction between time and type of area. Effect sizes were computed as partial eta squared values (η_p_^2^). Mauchly’s test indicated that the assumption of sphericity was violated in all general linear models with repeated measures options performed. Therefore, we corrected the degrees of freedom using the more conservative Greenhouse-Geisser correction (epsilon <0.75) [41].

The effect of wolf territory size on the number of harvested moose was examined using GLM repeated measures. Wolf territory size was classified as a dichotomous variable according to whether it was smaller or larger than the average wolf territory size (1,000 km^2^ using 100% MCP) [42]. Wolf territory size (small and large) was used as a fixed factor.

Next we tested if the number of harvested adult moose (males and females pooled) differed from the hunting quotas allocated in both wolf territories and control areas (analysed separately) using GLM repeated measures. Harvest and quotas were used as a fixed factor. The same analysis was conducted using quotas allocated as dependent variable and type of area as a fixed factor.

We tested if hunting effort differed between wolf territories and control areas using generalized linear mixed model (GLMM). This analysis enabled us to model variables measured at multiple time scales with an unbalanced design (due to voluntary reporting). We used a Poisson distribution for the dependent variable (counts of hours during a fixed time period). We included hunting team as a random intercept to account for multiple observations. We used type of area and year (continuous variable) as fixed factors.

Finally, we used a liner mixed model (LMM) to test which factors influenced the number of moose (total, males, females, calves, and calves per female) observed by hunters within wolf territories and control areas per hour spent hunting. This analysis enabled us to model variables measured at multiple time scales with an unbalanced design that in our case was caused by the requirement of at least 5,000 observation hours. We used management units as a random intercept to account for multiple measurements. Type of area and year were set as fixed factors.

### Calculations of Harvest Reductions Needed to Compensate for Wolf Predation

We modelled the required numerical reduction of hunter harvest and/or the composition of the hunted animals needed to compensate for wolf predation on an average territory size (1,000 km^2^). We used a sex- and age-structured model with 17 age classes for females and 13 age classes for males [18] of a moose population written in R (R Development Core Team 2012). The model included winter survival, fecundity, summer predation by wolves, autumn harvest by hunters, and winter predation by wolves. A thorough description of model structure, assumptions, parameter values, dynamics and relevance for our study system are available in Table 1 in Jonzen et al. [18]. The model quantifies harvest by both 1) the total number of moose shot and 2) the proportion of this total that consists of adult females. Including the proportion of shot animals that are females is important because adult females have the highest reproductive value and their removal has therefore a disproportionate impact on population size [43]. We used this model to estimate hunting strategies that would keep the moose population at a growth rate λ = 1 for 100 years, starting from a population at its asymptotic state. A sustainable harvest can be achieved by varying the total quota or a proportion of females in that quota. When total quota increases (and the proportion of females in the quota is not changed), the total number of shot females increases. When the proportion of females in the quota increases, the total number of shot animals does not vary because the increase of females in the quota is matched by a decrease of males and calves. This description of the harvest strategy properly model how hunters make quota-setting decisions: by varying total number and proportion of females in that total. We used the model to simulate moose populations with and without wolf predation and identify sustainable hunting strategies and then compared these simulated data with data from the 5+5 year areas and the 10-year areas. We could then assess whether changes in harvest by hunters were enough to compensate for the additional mortality on moose caused by wolf predation. Our model needed a starting moose density and we made the assumption that these densities were within ranges of values that would make the first year harvest sustainable (λ = 1). This assumption is arbitrary—the harvest could have been overharvesting—however our results are independent of this assumption because we are interested in relative changes in harvest and not in absolute values. Calculated initial moose densities were 11 ± 1 moose 10 km^-2^ for the 5+5 year wolf areas, 9 ± 1 moose 10 km^-2^ for the 5+5 year control areas, 16 ± 1 moose 10 km^-2^ for the 10-year wolf areas, and 11 ± 1 moose 10 km^-2^ for the 10-year control areas.

**Table 1:** Effects of time (ten-year periods) and type of area (wolf territories and control areas) on hunter harvest of moose (total number of moose harvested as well as number of males, females and calves), using a general linear model with the repeated measures options.

Area	Moose category	Variables	df	F	P
5+5 year	Total	**Time**	4.02, 192.80	18.95	**<0.001**
		Type	1, 48	0.04	0.947
		**Time × Type**	4.02, 192.80	3.90	**0.004**
	Male	**Time**	6.14, 294.93	5.42	**<0.001**
		Type	1, 48	0.015	0.904
		**Time× Type**	6.14, 294.93	1.69	0.121
	Female	**Time**	5.84, 280.35	12.54	**<0.001**
		Type	1, 48	0.34	0.561
		**Time × Type**	5.84, 280.35	4.08	**0.001**
	Calf	**Time**	5.31, 254.79	10.57	**<0.001**
		Type	1, 48	0.044	0.835
		Time × Type	4.64, 222.86	1.81	0.117
10-year	Total	**Time**	5.84, 490.23	40.85	**<0.001**
		**Type**	1, 84	35.96	**<0.001**
		**Time × Type**	5.84, 490.23	4.41	**<0.001**
	Male	**Time**	7.56, 634.65	13.46	**<0.001**
		**Type**	1, 84	22.50	**<0.001**
		Time × Type	7.56, 634.65	1.90	0.061
	Female	**Time**	6.66, 559.68	13.07	**<0.001**
		**Type**	1, 84	26.36	**<0.001**
		**Time × Type**	6.66, 559.68	3.43	**0.002**
	Calf	**Time**	6.77, 568.40	20.07	**<0.001**
		**Type**	1, 84	21.96	**<0.001**
		**Time × Type**	6.77, 568.40	2.08	**0.046**

Analyses were conducted for areas five years prior to wolf establishment and five years with presence of wolves, and areas with continuous wolf presence during ten years.

## Results

In total, including all areas, 31,827 moose (30% males, 26% females and 44% calves) were harvested during the study period within a land area of 8,440–8,780 km^2^.

### Harvest, Quotas and Effort in 5+5 Year Areas

**Fig 1:**
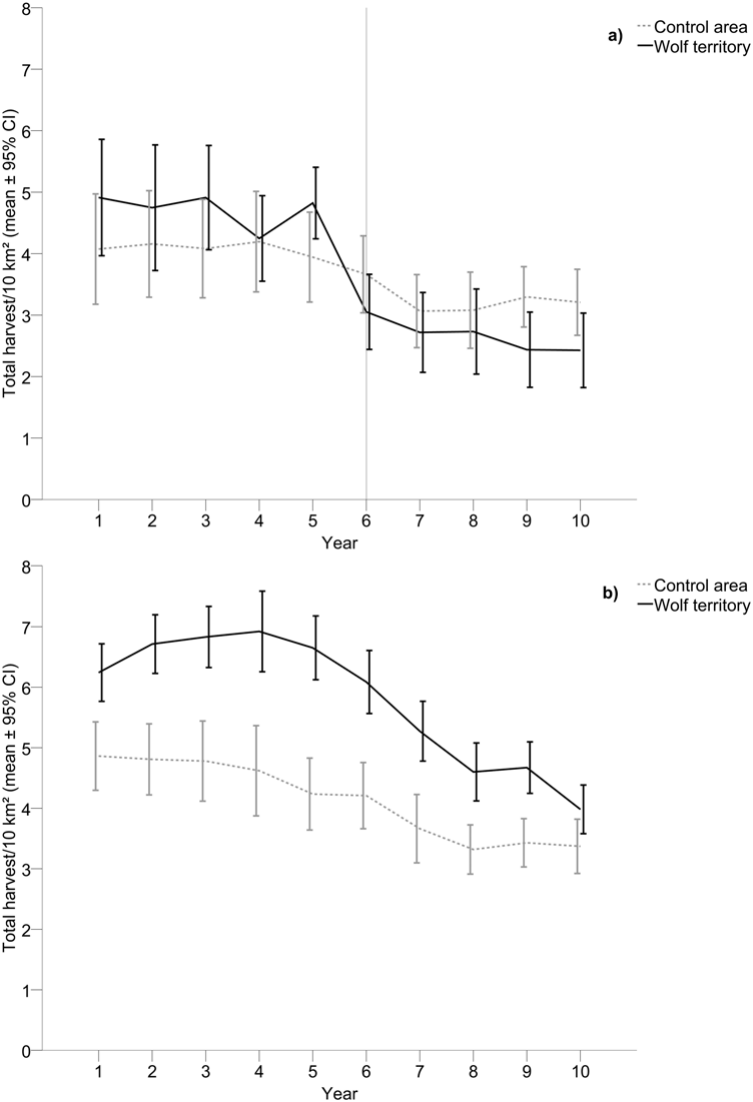
Harvest of moose within wolf territories compared to control areas. Total harvest of moose (males, females and calves pooled) during a) five years prior to wolf establishment and five years with wolf presence (n = 25) compared to control areas, and b) ten years with presence of wolves (n = 43) compared to control areas. The vertical line in a) indicates the first year with wolf presence.

The total number of harvested moose did not differ between wolf territories (mean ± 95% CI = 3.70 ± 0.26) and control areas (3.68 ± 0.22), but decreased with time (mean_year 1_ = 4.49 ± 0.64, mean_year 10_ = 2.82 ± 0.41, B = -0.24 ± 0.055, Table 1, Fig. 1a). This pattern was also evident for all single categories of moose (Table 1). A statistically significant interaction effect between time and type of area (wolf territories and control areas, Table 1) for the total number of harvested moose and for females separately showed that the reduction in harvest was larger within wolf territories compared to control areas (Fig. 1a). As the reduction in harvest within wolf territories compared to control areas was mainly a result of a decreased number of females harvested, the composition of the hunted animals changed after wolf establishment. The largest effect between consecutive years occurred between year five and year six (the first year with presence of wolves) for both the total number of harvested moose (F_1, 48_ = 14.05, p < 0.001, η_p_^2^ = 0.23, Fig. 1a) and for females separately (F_1, 48_ = 6.71, p = 0.013, η_p_^2^ = 0.12).

**Fig 2:**
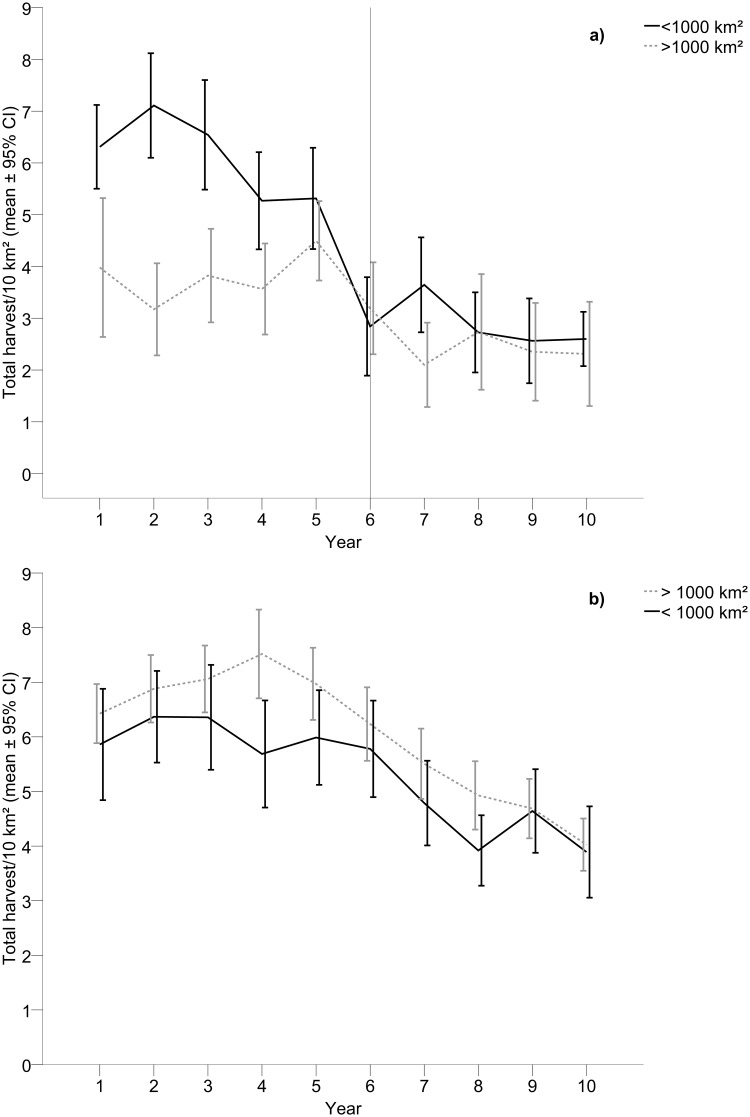
Harvest of moose inside small and large wolf territories. Total harvest of moose (males, females and calves pooled) during a) five years prior to wolf establishment and five years with wolf presence (n = 25), and b) ten years with presence of wolves (n = 43) in relation to small and large wolf territories. The vertical line in a) indicates the first year with wolf presence.

The reduction in the total number of moose harvested after wolf establishment was larger within small (range 500–800 km^2^) wolf territories, compared to large (range 1,200–1,800 km^2^) wolf territories (Table 2, Fig. 2a). This pattern was also found for the single categories of males and calves, and showed a tendency to decrease also for females (p = 0.076, Table 2).

**Table 2:** Effects of time (ten-year periods) and wolf territory size (<1,000 km^2^ or >1,000 km^2^) on hunter harvest of moose (total number of moose harvested as well as number of males, females and calves).

Area	Moose category	Variables	df	F	P
5+5 year	Total	**Time**	5.17, 118.84	22.59	**<0.001**
		**Wolf territory size**	1, 23	9.96	**0.004**
		**Time × Wolf territory size**	5.17, 118.84	7.25	**<0.001**
	Male	**Time**	5.29, 121.67	5.46	**<0.001**
		**Wolf territory size**	1, 23	16.65	**0.017**
		**Time × Wolf territory size**	5.29, 121.67	3.66	**0.003**
	Female	**Time**	5.26, 121.01	12.25	**<0.001**
		**Wolf territory size**	1, 23	14.42	**0.001**
		Time × Wolf territory size	5.26, 121.01	2.03	0.076
	Calf	**Time**	5.05, 116.16	12.32	**<0.001**
		Wolf territory size	1, 23	1.65	0.212
		**Time × Wolf territory size**	5.05, 116.16	5.38	**<0.001**
10-year	Total	**Time**	5.82, 238.47	26.73	**<0.001**
		Wolf territory size	1, 41	3.79	0.059
		Time × Wolf territory size	5.82, 238.47	1.61	0.147
	Male	**Time**	6.64, 272.12	9.20	**<0.001**
		Wolf territory size	1, 41	3.71	0.061
		Time × Wolf territory size	6.64, 272.12	1.54	0.157
	Female	**Time**	6.28, 257.55	9.27	**<0.001**
		Wolf territory size	1, 41	3.05	0.088
		Time × Wolf territory size	6.28, 257.55	1.12	0.351
	Calf	**Time**	6.11, 250.54	12.97	**<0.001**
		Wolf territory size	1, 41	0.87	0.358
		Time × Wolf territory size	6.11, 250.54	0.77	0.594

A general linear model with the repeated measures options was used. Analyses were conducted for areas within wolf territories during five years prior to wolf establishment and five years with presence of wolves, and areas with continuous wolf presence during ten years.

**Fig 3:**
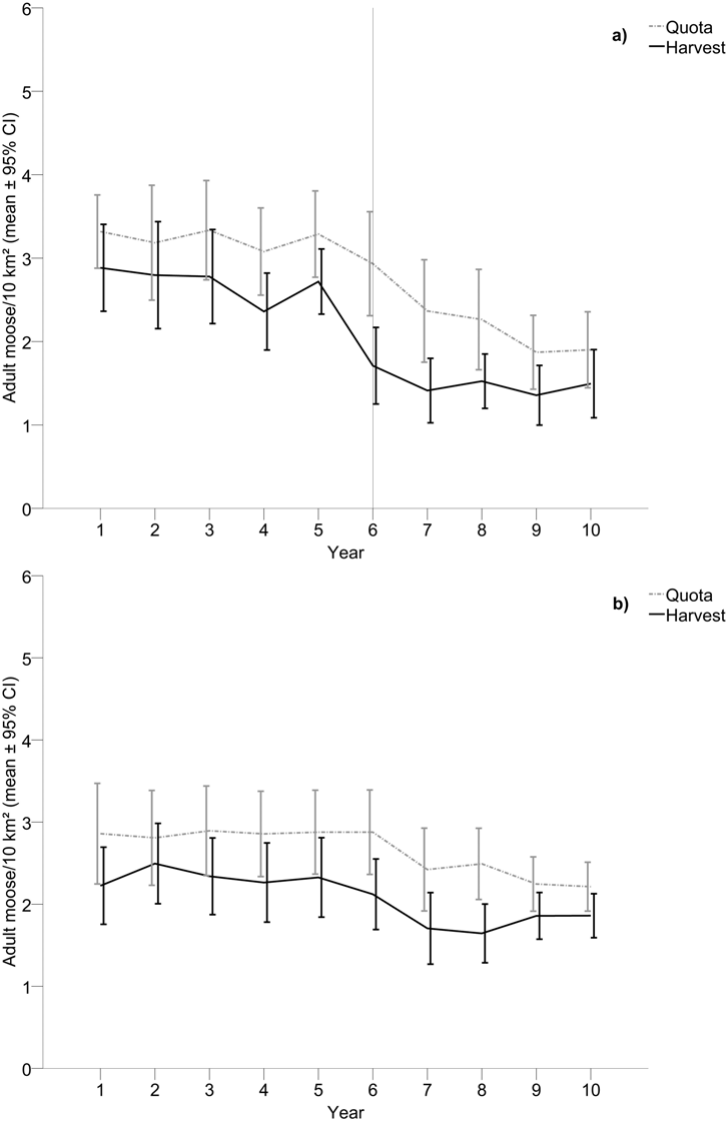
Quota and harvest of adult moose within wolf territories compared to control areas. Quota and hunter harvest of adult moose (males and females pooled) within a) wolf territories (n = 22) and b) control areas during five years prior to wolf establishment and five years with wolf presence. The vertical line in a) indicates the first year with wolf presence.

Quotas allocated for adult moose (2.75 ± 0.18) were higher than the actual harvest (2.10 ± 0.16). There was no statistically significant interaction with time (Table 3, Fig. 3a) indicating that the actual harvest followed the same trend as the quotas allocated over the study period. The largest difference between quotas and harvest during the 10-year period occurred between year five and year six (the difference increased by 110%, F_1, 42_ = 6.23, p = 0.017, η_p_^2^ = 0.13). Similarly, for control areas the quotas allocated were higher (2.65 ± 0.14) than actual hunter harvest (2.08 ± 0.13), and showed no interaction with time (Table 3, Fig. 3b), but with the largest difference between years occurring between year eight and year nine (the difference decreased by 54%, F_1, 42_ = 4.81, p = 0.034, η_p_^2^ = 0.10). Comparing quotas between wolf territories and control areas showed that there were no difference (Table 4) but that quotas decreased with time (mean_year 1_ = 3.09 ± 0.37, mean_year 10_ = 2.01 ± 0.27, Table 4, Fig. 3a-b). There was a tendency of a statistically significant interaction with time (p = 0.055, Table 4) indicating that quotas decreased with time in wolf territories but remained similar in control areas. A comparison of quota development with time between wolf territories and control areas showed that the largest difference between consecutive years occurred between year five and year six where quotas remained similar in control areas but was reduced in wolf territories (F_1, 42_ = 5.39, p = 0.025, η_p_^2^ = 0.11).

**Table 3:** Effects of time (ten-year periods) on quotas allocated and actual hunter harvest of adult moose (males and females pooled), using a general linear model with the repeated measures options.

Area	Type	Variables	df	F	P
5+5 year	Wolf	**Time**	4.50, 188.80	23.65	**<0.001**
		**Quota and Harvest**	1, 42	6.63	**0.014**
		Time× Quota and Harvest	4.50, 188.80	1.13	0.346
	Control area	**Time**	3.50, 146.83	9.92	**<0.001**
		**Quota and Harvest**	1, 42	4.59	**0.038**
		Time × Quota and Harvest	3.50, 146.83	1.05	0.381
10-year	Wolf	**Time**	4.79, 354.43	52.51	**<0.001**
		**Quota and Harvest**	1, 74	36.41	**<0.001**
		**Time × Quota and Harvest**	4.79, 354.43	9.62	**<0.001**
	Control area	**Time**	4.93, 364.48	12.97	**<0.001**
		**Quota and Harvest**	1, 74	26.30	**<0.001**
		**Time × Quota and Harvest**	4.93, 364.48	2.81	**0.017**

Analyses were conducted for areas five years prior wolf establishment and five years with presence of wolves, and in areas with continuously wolf presence during ten years, as well as in their respectively control areas.

**Table 4:** Effects of time (ten-year periods) and type of area (wolf territories and control areas) on quotas allocated of adult moose (males and females pooled), using a general linear model with the repeated measures options.

Area	Variables	df	F	P
5+5 year	**Time**	3.15, 132.48	18.01	**<0.001**
	Type	1, 42	0.110	0.742
	Time × Type	3.15, 132.48	2.56	0.055
10-year	**Time**	3.62, 267.72	40.50	**<0.001**
	**Type**	1, 74	31.58	**<0.001**
	**Time × Type**	3.62, 267.72	6.33	**<0.001**

Analyses were conducted for areas five years prior to wolf establishment and five years with presence of wolves, and in areas with continuous wolf presence during ten years.

Also hunting effort decreased with time (median_year 1_ = 581, median_year 10_ = 329), but the rate of decrease in hunting effort did not differ between wolf territories and control areas (Table 5).

**Table 5:** Parameter values (B) of year and type of area (wolf territories and control areas) on hunting effort, using a generalized linear mixed model.

Area	Variables	B	SE	P	Odds ratio	95% CI for odds ratio	
						Lower	Upper
5+5 year	**Year**	-0.050	0.025	**0.044**	0.951	0.906	0.999
	Control area	0.381	0.352	0.280	1.464	0.733	2.924
	Wolf territory	0	0				
	Year × Control area	0.011	0.032	0.722	1.011	0.950	1.076
	Year × Wolf territory	0	0				
10-year	Year	-0.010	0.008	0.210	0.990	0.975	1.006
	Control area	-0.097	0.169	0.565	0.908	0.652	1.264
	Wolf territory	0	0				
	Year × Control area	0.003	0.011	0.758	1.003	0.982	1.026
	Year × Wolf territory	0	0				

Analyses were conducted for areas five years prior wolf establishment and five years with presence of wolves, and in areas with continuously wolf presence during ten years.

### Harvest, Quotas, Effort and Observations in 10-year Areas

For the 10-year areas the total number of harvested moose was higher in wolf territories (5.80 ± 0.18) compared to control areas (4.13 ± 0.18) and decreased with time (mean_year 1_ = 5.55 ± 0.39, mean_year 10_ = 3.68 ± 0.30, B = -0.26 ± 0.046, Table 1, Fig. 1b). The same pattern of harvest occurred for all single categories of moose (Table 1). The statistically significant interaction effect between time and type of area (wolf territories and control areas, Table 1) for the total number of moose (Fig. 1b), females separately and calves separately, as well as the close to statistically significant effect for males separately (p = 0.061) showed that the reduction in harvest was larger within wolf territories compared to control areas. The harvest of the total number of moose (Fig. 2b), males separately, and females separately tended (p = 0.059–0.088) to be lower in small territories (range 900–1,000 km^2^), compared to large territories (range 1,100–2,000 km^2^, Table 2).

**Fig 4:**
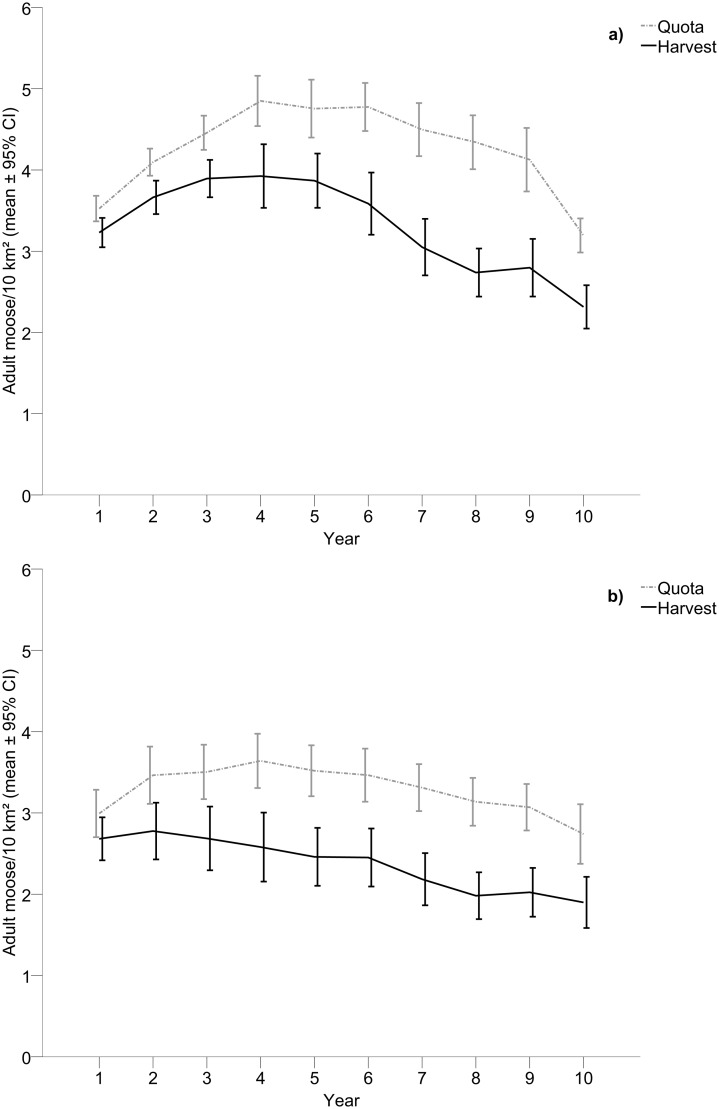
Quota and harvest of adult moose within wolf territories compared to control areas. Quota and hunter harvest of adult moose (males and females pooled) within a) wolf territories with at least ten years of presence of wolves (n = 38) and b) adjacent control areas.

Quotas allocated were higher within wolf territories (4.26 ± 0.10) than actual harvest (3.31 ± 0.11) and this difference increased with time (Table 3, Fig. 4a). The same pattern was found in control areas with higher quotas (3.28 ± 0.10) than actual harvest (2.37 ± 0.11) and again this difference increased with time (Table 3, Fig. 4b). Consequently, unlike 5+5 year areas, the actual harvest in 10-year areas diverged more from the quotas allocated with time in both wolf territories and control areas. Similar to harvest, quotas allocated differed between wolf territories and control areas and the interaction effect showed that quotas were reduced more with time within control areas compared to wolf territories (Table 4, Fig. 4a-b).

Contrary to the 5+5 year areas hunting effort did neither change with time nor differ between wolf territories and control areas (Table 5).

The total number of observed moose per hour was higher within wolf territories (0.066 ± 0.006) compared to control areas (0.058 ± 0.005) and decreased with time (Table 6). The same pattern was shown for the number of observed females (0.029 ± 0.003 within wolf territories and 0.024 ± 0.002 in control areas, Table 6). In contrast, the number of observed males and calves per hour did not differ between wolf territories and control areas but decreased with time for calves, but not for males (Table 6). The composition of age and sex classes in the moose populations during the ten-year period was similar within wolf territories and control areas, as there was no significant interaction effect for any of the moose categories (Table 6). However, the number of calves per female was significantly lower (16%) within wolf territories (0.63 ± 0.03) compared to control areas (0.75 ± 0.04, Table 6) and this did not change with time either within wolf territories or in control areas.

**Table 6:** Parameter values (B) of the main effect of year (ten-year periods) and type of area (wolf territories and control areas), as well as their interaction, on the number of moose observed by hunters during the first week of the harvest (total number of moose, females, calves, and calves per female).

Moose category	Variables	B	SE	df	t	P
Total	**Year**	-0.0026	0.0007	19.255	-3.559	**0.002**
	**Control area**	-0.0172	0.0073	32.195	-2.372	**0.024**
	Wolf territory	0	0			
	Year × Control area	0.00167	0.0010	19.255	1.627	0.120
	Year × Wolf territory	0	0			
Male	Year	-1.1×10^-10^	9.8×10^-8^	2×10^15^	-0.001	1.000
	Control area	-7.2×10^-10^	1.1×10^-6^	2×10^15^	-0.001	1.000
	Wolf territory	0	0			
	Year × Control area	9.1×10^-11^	1.4×10^-7^	2×10^15^	0.001	1.000
	Year × Wolf territory	0	0			
Female	**Year**	-0.00109	0.0004	25.686	-3.109	**0.005**
	**Control area**	-0.00663	0.0030	31.421	-2.220	**0.034**
	Wolf territory	0	0			
	Year × Control area	0.00044	0.0004	25.686	0.884	0.385
	Year × Wolf territory	0	0			
Calf	**Year**	-0.00123	0.0004	31.246	-3.325	**0.002**
	Control area	-0.00130	0.0036	32.076	-0.363	0.719
	Wolf territory	0	0			
	Year × Control area	0.00052	0.0005	31.246	0.988	0.331
	Year × Wolf territory	0	0			
Calves per female	Year	-0.00895	0.0053	26.555	-1.687	0.103
	**Control area**	0.10332	0.4636	21.332	2.229	**0.037**
	Wolf territory	0	0			
	Year × Control area	0.00252	0.0075	26.555	0.335	0.740
	Year × Wolf territory	0	0			

A linear mixed model was used. Analyses were conducted in areas with continuous wolf presence during ten years.

### Reduction in Harvest to Compensate for Wolf Predation

**Fig 5:**
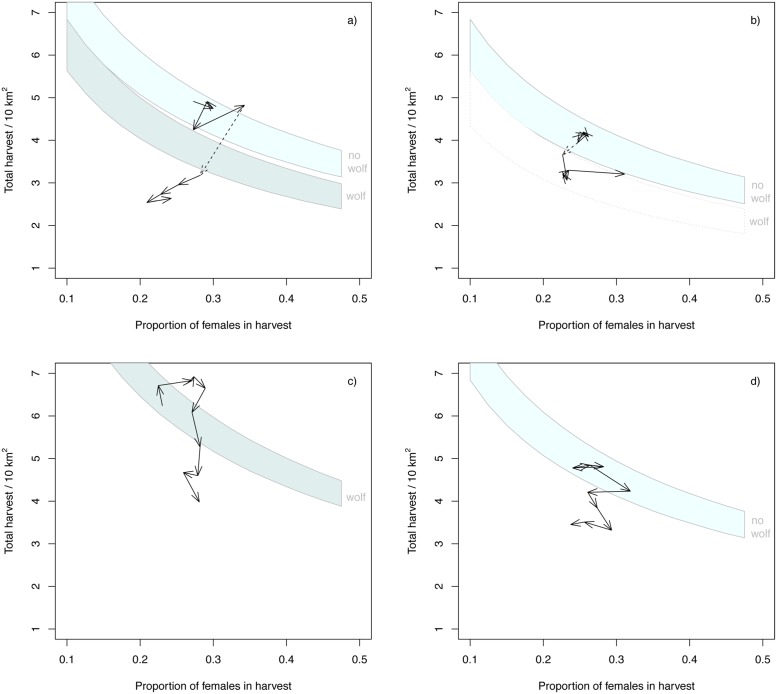
Simulated and realized changes of moose harvest within wolf territories compared to control areas. The x axis indicates the proportion of females harvested and the y axis indicates the total number of moose harvested. The continuous bands indicate values of harvest rates and proportions of females in harvest (light grey: without wolves, dark grey: with wolves) calculated assuming a moose density that would make sustainable the first year harvest. The arrows indicate the yearly averages of annual total harvest and the proportion of females in this harvest in a) 5+5 year wolf areas, b) 5+5 year control areas, c) 10-year wolf areas, and d) 10-year control areas. The dotted line in a) shows the year when wolves returned and in b) the comparison if wolves had established.

Data showed that hunters reduced the total number of harvested moose (Fig. 5). In the 5+5 year wolf areas the actual average reduction in the total number of moose harvested was 2.1 moose 10 km^-2^ during the first five years after wolf establishment including a reduction of 1/3 of the proportion of females. This reduction was higher than the estimated reduction needed to compensate for wolf predation (Fig. 5a). Harvest in the 5+5 year control areas also declined but not to an extent that would match the amount theoretically required compensating fully for wolf predation (Fig. 5b), suggesting that the decline in the 5+5 wolf areas was mainly driven by wolf establishment. In the 10-year areas, harvest declined both within wolf territories (Fig. 5c) and in the control areas (Fig. 5d), with the former experiencing a much larger decline.

## Discussion

Hunter harvest of moose decreased whether or not within wolf territories. The reduction in hunter harvest of moose was stronger within wolf territories compared to control areas in line with our predictions. This pattern was evident both during the phase of wolf establishment as well as in areas where wolves had been present continuously during at least ten years. The decrease in hunter harvest with time also in control areas most likely indicated a general decline in the moose population, and was also indicated by data from hunters' observations. Density dependent harvesting, has also has been reported for Norwegian moose populations [4, 44, 45]. The general decline found in moose harvest in this study in both wolf territories and control areas is likely a result of a large-scale intentional management strategy, which has been to reduce damage to commercially valuable forest trees by moose.

This study area is unique in terms of the history of the high harvest rates of moose in combination with an expanding wolf population. Our results add up to several previous studies in other areas in the world discussing the need for a reduction in hunter harvest after establishment of an additional predator. In Alaska, USA, where moose have been continuously exposed to predation by wolves and/or bears (brown bear and black bear [*Ursus americanus*]), harvest yields increased in areas with predator control [46]. Similarly, in the Greater Yellowstone Area (GYA), USA, which has a newly established and expanding wolf population, both empirical data and theoretical modelling supported a necessary reduction in hunter harvest of elk (*Cervus elaphus*) to prevent a decline in elk numbers [47, 48, 49]. In contrast, it has been argued that wolf predation of elk after the reintroduction of wolves to GYA was largely compensatory and were not the underlying cause of the decline in the elk population after the return of wolves [6]. Instead, it was suggested that the decline could be caused by changes in climatic conditions in combination with an increase in the hunter harvest outside the park [6]. In Michigan, Minnesota and Wisconsin, USA, the white-tailed deer (*Odocoileus virginianus*) populations exists at high densities and has been relatively little affected by predation despite a strong recovery of wolves [50]. The authors' explanation for the lack of impact of wolves on these prey populations was the high reproductive potential characterizing white-tailed deer in combination with mild winters.

The Scandinavian moose population have a very high reproductive potential [51] and wolf predation is strongly selective in favour of calves and old females [18, 20, 31]. Thus, prey selection by wolves is in strong contrast to hunter harvest which is biased towards a higher (than wolves’) proportion of adults and male-biased for the adult segment [11, 52]. In long-lived species such as moose, animal age and sex classes generally contribute differently to population growth by having different reproductive values [13, 53]. Therefore, hunter harvest will have a stronger impact on moose population growth per unit kill. Also, a significant part of the wolves’ predation target calves early in the summer that in turn may relieve females from the cost of lactation [22]. Wolf predation may therefore, unlike hunter harvest which is mainly performed during fall [37], to some extent result in reproductive compensation among adult female moose. However, even if this compensatory mechanism was present in the Swedish moose population, hunter observations of moose indicate that this was not enough to fully compensate for the combined effect of wolf predation and hunter harvest.

In our study, hunters responded (as quotas only specify the permitted number of adults to be harvested) to the establishment of wolves not only by adjusting the total size of harvest but also by changing the composition of the hunted animals by lowering the proportion of females in harvest. This reduction in female harvest may to a certain extent compensate for a lower numerical reduction of harvest than the estimated loss from wolf predation [11, 18]. In GYA, it has been argued that an increase in female survival of elk is necessary to stop the declining numbers of elk resulting from the combined effects of hunter harvest and predation [48]. Besides the reduction of the female harvest in the Swedish moose population, the reduction in the number of harvested calves found may either be an intentional strategy by hunters to compensate for, or be a consequence of the high predation pressure by wolves on calves during summer [20]. Data from hunters' observations indicated that the latter explanation have more support since the hunters both harvested and observed fewer calves per female within wolf territories. Interestingly, the harvest strategy mainly involving a reduction of females and calves, as shown within areas with wolf presence during at least ten years, resulted in a similar sex and age structure (as derived from moose observations) of the moose populations to that found within control areas. However, what is worth noting is that the moose harvest in wolf territories either remained higher (10-year areas) or were similar (5+5 year areas) than in control areas.

Our results showed mixed support for that an adaptive moose management strategy involving both managers (hunting quotas) and hunters (the actual harvest) were applied after wolf establishment. In this context, we use the term adaptive management as a deliberate strategy realized by both hunters and managers to adjust harvest size and/or composition to not further reduce moose density (and the future sustainable yield). Two observations support an adaptive moose management strategy in the 5+5 year areas. First, both decreased quotas and the reduction in actual harvest was sufficient to compensate both for wolf predation and for the general downward trend in size of the moose populations as indicated in control areas. Second, the most pronounced reduction in actual harvest and hunting quotas was evident in the first year after wolf establishment, demonstrating an instant response by both managers and hunters to the establishment of an additional predator. The rapid behavioural response by managers and hunters after the establishment of a new predator in our study area also contrasts with the more commonly observed time-lagged functional response between hunter harvest rates and changes in ungulate population densities [4, 54]. Consequently, the rapid response observed in our study was likely more a result of an anticipated increase in moose mortality triggered by the information on wolf establishment than by a biological functional response linked to actual changes in moose population density. However, a functional response by managers and hunters due to a reduction in moose population density may have been apparent in the 10-year areas since the harvest size here showed a larger reduction with time compared to control areas. This overexploitation hypothesis is also supported by data on hunter observations of moose which showed a reduction over time in the number of moose observed. Interestingly, this negative trend in the number of moose observed did not differ between wolf territories and control areas indicating that wolf predation may not have been the main causal mechanism to this trend.

One prerequisite for the hunters to respond adaptively is that the establishment of new wolf territories are correctly monitored and communicated to the hunter community at an early stage of wolf territory establishment. In Sweden, all County Administrative Boards are obliged to census and report newly established wolf territories and estimate their approximate size and geographical distribution each year. With this knowledge managers and hunters have the option to respond and adjust the harvest quota accordingly. The management of moose in Sweden contrasted to the situation in GYA, where hunter harvest of elk was not initially reduced with wolf reintroduction, but was gradually reduced over time [55].

Information only on presence or absence of wolves is not enough for a successful moose management. We showed that smaller wolf territories were correlated with a relatively larger reduction in hunter harvest as compared to larger territories. Wolf territory sizes decrease when a growing population becomes saturated [56]. An alternative explanation for decreasing wolf territory sizes is increasing prey densities [57]. However, such a relationship between territory size and wolf density or moose density has not been confirmed on the Scandinavian Peninsula [42]. Also, there is evidence that wolf predation rates are strongly related to the moose-wolf ratio [58] or the abundance of moose within wolf territories [59] which in turn is positively related to wolf territory size. Therefore, in small territories, wolves exert a higher predation rate than in large territories which results in a lower sustainable harvest.

Wolf territories with at least ten years of wolf presence (10-year areas) represent the parts of Scandinavia first colonized by wolves. Interestingly, our data showed that both initial harvest and moose observations made by hunters within first colonized territories were higher than in both control areas and areas later colonized by wolves (5+5 year areas). This indicates that wolves on the Scandinavian Peninsula during early colonization selected high moose density areas for territory establishment and that even a decade after wolf establishment, moose densities were still high enough to result in a larger harvest relative to control areas.

Our findings may have important implications for the generality of ecosystem effects that may be anticipated from the colonization by large predators into new or formerly inhabited areas. Many studies on ecosystem effects of large predators have so far been directed to areas with little or no human impact [60]. Re-colonization of large predators into these areas have been suggested to result in strong ecosystem effects including trophic cascades, partly as a result of direct numerical effects on prey density [61]. In this study of a system under strong human influence we show that the re-colonization of wolves resulted in an almost instant functional response by another large predator—humans—that precluded or at least reduced a direct numerical effect on the density of wolves’ main prey, the moose. Because most of the worlds’ habitat that will be available for future colonization by large predators are likely to be strongly influenced by humans (similar to our study area), human response behaviour may constitute an important factor that ultimately may govern the impact of large predators on their prey and thus on potential trophic cascades.
